# Large difference in carbon emission – burial balances between boreal and arctic lakes

**DOI:** 10.1038/srep14248

**Published:** 2015-09-15

**Authors:** E. J. Lundin, J. Klaminder, D. Bastviken, C. Olid, S. V. Hansson, J. Karlsson

**Affiliations:** 1 Department of Environmental Science and Analytical Chemistry (ACES), Stockholm University, SE-106 91 Stockholm, Sweden; 2 Department of Ecology and Environmental Science, Umeå University, SE-90187 Umeå, Sweden; 3 Department of Thematic Studies–Environmental Change, Linköping University, SE-58183 Linköping, Sweden; 4 Université de Toulouse; INP, UPS; EcoLab; ENSAT, Avenue de l’Agrobiopole, 31326 Castanet Tolosan, France; 5 CNRS; EcoLab; 31326 Castanet Tolosan, France; 6 Climate Impacts Research Centre (CIRC), Department of Ecology and Environmental Science,Umeå University, SE-981 07 Abisko, Sweden

## Abstract

Lakes play an important role in the global carbon (C) cycle by burying C in sediments and emitting CO_2_ and CH_4_ to the atmosphere. The strengths and control of these fundamentally different pathways are therefore of interest when assessing the continental C balance and its response to environmental change. In this study, based on new high-resolution estimates in combination with literature data, we show that annual emission:burial ratios are generally ten times higher in boreal compared to subarctic – arctic lakes. These results suggest major differences in lake C cycling between biomes, as lakes in warmer boreal regions emit more and store relatively less C than lakes in colder arctic regions. Such effects are of major importance for understanding climatic feedbacks on the continental C sink – source function at high latitudes. If predictions of global warming and northward expansion of the boreal biome are correct, it is likely that increasing C emissions from high latitude lakes will partly counteract the presumed increasing terrestrial C sink capacity at high latitudes.

One of the big challenges of our time is to understand greenhouse gas dynamics in order to evaluate its effect on climate change^1^. Understanding the climate system requires knowledge of climatic effects on global C cycling, including the magnitude and control of various sources and sinks in coupled land – water – atmospheric systems. High latitude regions are of special interest in a future climate warming scenario, as the surface air temperature increase is predicted to be amplified towards northern high latitudes where sensitive ecosystems may experience significant change and exert strong feedback effects on the climate system^2^. It is now clear that aquatic systems are of major significance in the C cycle, being considered as large atmospheric sources of CO_2_ and CH_4_^3^,^4^. Globally, lake emissions exceed the continental lateral C export and represent about 20% of the oceans CO_2_ sequestration^3^,^4^,^5^. Simultaneously, inland waters have been recognized to bury significant amounts of C in sediments where it accumulates over geological time-scales^6^,^7^,^8^. The organic C burial in inland water sediments is three-fold higher than the burial in ocean sediments, making inland water sediments comparable to the C stocks of northern peatlands, soils and biomass combined, and constitutes the second to third largest C pool in northern environments^6^,^7^,^8^. At high latitudes, lakes cover a substantial part of the land area^5^,^9^,^10^. For example, latitudes of 60°–69° N contain 24% of the global lake area^9^, making northern lakes important components of the global C cycle^10^.

Despite their importance in the global C cycle, the knowledge about northern lake C emissions relative to burial rates is poor. Although it is known that boreal lakes are generally more supersaturated in CO_2_ than subarctic-arctic lakes^11^, very few field studies have quantified burial and atmospheric exchange simultaneously in northern lakes for cross comparison between biomes. Especially, the understanding of the emission – burial balance within subarctic – arctic lakes represents a weak point in the literature (see Supplementary Information) which prevents cross climate zone comparisons with e.g. boreal lakes^12^. In this study, we combine new detailed measurements of annual C emission as well as annual C burial in six subarctic - arctic lakes in northern Sweden with literature data to compare these fluxes across biomes.

## Results and Discussion

Total annual C emissions (CO_2_ + CH_4_) from the investigated subarctic lakes ranged between 5 and 54 g C m^−2^ yr^−1^. Overall, the C emissions were dominated by CO_2_ which accounted for more than 90% of the total annual C emission in all lakes except one, where CO_2_ emission was low and CH_4_ accounted for 40% of the annual C emission. The dissolved CO_2_ and CH_4_ accumulated during winter under ice and released at ice break-up constituted between 7 to 80% of annual emissions. The burial of C varied between 5 and 25 g C m^−2^ yr^-1^. We were not able to detect any significant mass loss in any of the sediment samples after acidification, indicating negligible accumulation of inorganic C in the sediments of our sampled lakes.

**Figure 1:**
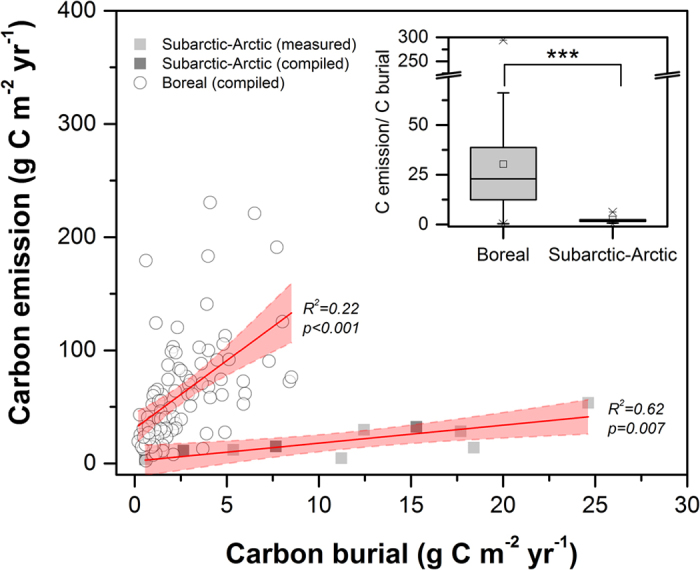
Linear relationships between paired C emissions and sediment burial in boreal (open circles) and subarctic – arctic (squares) lakes, with the regression 95% confidence intervals (in red shading). Black squares represent new data collected in this study. Grey squares and open circles represent data from literature, compiled for this study. The inset show the differences in emission:burial ratios for the same data set (F_1,97_ = 94.9, ***P < 0.001). The box corresponds to the 25^th^ and 75^th^ percentile, while the whiskers indicate 5^th^ and 95^th^ percentiles. The square corresponds to the arithmetic mean and the horizontal line the median. Outliers are indicated by crosses. All data sources are listed in the Supporting Information (Table S4).

**Figure 2:**
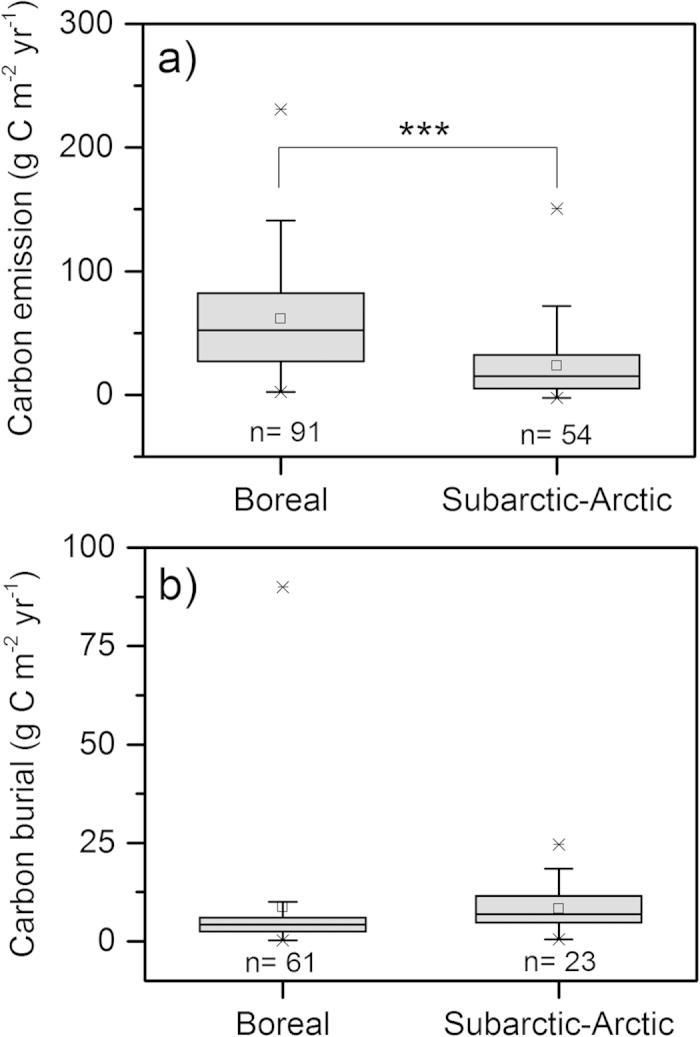
Published data and data from this study showing (**a**) deviating (F_1,143_ = 29.7, ***P < 0.001) C emission but (**b**) no significant difference in sediment C burial between boreal and subarctic – arctic lakes. Both paired and separate emission and burial data is used. The box corresponds to the 25^th^ and 75^th^ percentile, while the whiskers indicate 5^th^ and 95^th^ percentiles. The square corresponds to the arithmetic mean and the horizontal line the median. Outliers are indicated by crosses. All data sources are listed in the Supporting Information (Table S2 and S3).

Our original data combined with annual C emission rates and sediment burial rates for individual lakes from previously published studies (altogether 89 boreal and 10 subarctic – arctic lakes, see Table S4 in Supplementary Information) clearly show that the average ratio of emission to burial was substantially higher (F_1,97_ = 94.9, P < 0.001) in boreal lakes (34 ± 37; mean ± standard deviation) than in subarctic – arctic lakes (2.4 ± 1.7; mean ± standard deviation). In accordance with findings by Kortelainen *et al.*^12^, we found a weak linear relationship between C emissions and burial rates in boreal lakes (R^2^ = 0.27, P < 0.001). Furthermore, our data suggested that a linear relationship (R^2^ = 0.62, P = 0.007) also occurs in subarctic – arctic systems, but there the regression slope is less steep (F_95,96_ = 58.3, P < 0.001) compared to in boreal lakes due to a low emission:burial ratio (Fig. 1). By also including available emission and burial estimates derived from separate lakes (Table S2 and S3) in the analysis, our result reveals that boreal lakes have significantly higher C emissions than subarctic – arctic lakes (F_1,143_ = 29.7, P < 0.001, Fig. 2a), while C burial rates are comparable across biomes (Fig. 2b). Even though our study is based on data collected from a limited numbers of lakes, our results are supported by other studies that found similar differences in CO_2_ partial pressures between biomes^11^.

The consistent differences between boreal and subarctic – arctic lakes indicate that the C emission:burial ratio is related to the biome-specific lake characteristics. Emission and burial of C are intimately linked, as increased C emission may have a direct and counteracting effect upon the sediment supply rate of C^7^. A different average emission:burial ratio for lakes from different regions, as seen in our study (Fig. 1), is therefore not surprising but implies that the partitioning of C within lakes can be predicted from their current biome at an extent that has not previously been considered. Our results are based on a comparative study, but current limnological knowledge can be used to decipher the most likely mechanisms behind the systematic differences in emission:burial ratios between boreal and subarctic – arctic regions. Terrestrial productivity, and lateral organic C export, is higher in boreal compared to subarctic – arctic regions^13^,^14^,^15^,^16^. The resulting generally more-colored and organic C rich waters in boreal compared to subarctic – arctic lakes, are known for enhancing heterotrophic respiration of terrestrial organic C and for suppressing within-lake fixation of CO_2_^14^,^17^, and thus result in higher relative net CO_2_ production and C emission in boreal compared to subarctic – arctic lakes^15^,^16^. The higher water temperatures in boreal lakes further stimulate heterotrophic respiration rates and thus CO_2_ losses^18^,^19^, while the positive effect of warming on CO_2_ fixation is expected to be weaker as fixation is often constrained by poor light conditions or availability of nutrients^20^,^21^. Regional differences in nutrient availability (e.g. nitrogen and phosphorous) must be of subordinate importance for the patterns observed, given that the generally higher nutrient levels in boreal lakes stimulate CO_2_ fixation and net ecosystem production. This would rather decrease the net CO_2_ production^12^,^17^,^21^ and increase the accumulation of C in sediments, i.e. a pattern opposite to what we found. Hence, higher C emission in relation to burial in boreal lakes, as a result of more-colored and warm water in comparison to subarctic – arctic systems seems rational. The ratio of emission to burial can therefore not be assumed to be constant but instead exhibits pronounced differences among biomes, presumably linked to climate controlled catchment characteristics, such as water temperature and terrestrial export of organic matter. Although challenging, a firm mechanistic understanding of climate and catchment control of the C sink – source function of lake ecosystem requires long term and large scale experimentation^22^.

The results of this study are important for assessment of the role of lakes in the continental C balance. The arctic basin presently shows a negative C balance of 63 Tg C yr^−1^ 23 and up-scaling our results to the arctic region between 63°–90° N (See Supplementary Information) shows that presently lakes alone are atmospheric sources of about 30 ± 30 Tg C yr^−1^; hence, an increase in the emission:burial ratio has the capacity to greatly alter the C balance of the whole arctic. Climate models consistently foresee increased temperatures^24^, prolonged growing seasons^24^, and a northward expansion of the tree and shrub cover at a pan-arctic scale^25^,^26^,^27^. An expansion of the climatic conditions currently prevailing in the boreal biome, into the arctic would convert a significant portion of current subarctic – arctic lakes into warmer and more colored lakes^14^,^15^,^28^. Our data suggest that this would lead to strongly enhanced lake C emissions while no increase in burial rates can be expected. If also taking the higher emissions of CH_4_ from boreal lakes, and its strong greenhouse forcing potential into account, we suggest that these effects work to counteract the potential increased terrestrial C sink capacity that potentially follows a warmer climate^3^,^29^,^30^. At the same time, there are minor differences in partial CO_2_ pressures between boreal and cold temperate lakes^11^, indicating small changes in lake emissions following a southern regression of the boreal biome. Considering the importance of aquatic systems for the high latitude continental C balance^5^,^16^,^31^, our results thus reveal a long-term unforeseen climate feedback; regardless of whether terrestrial C sequestration is favored by the future climate or not, lakes’ direct and indirect response to an expansion of the boreal biome weakens the northern inland C sink. We therefore conclude that the understanding of lakes’ sink – source functions will be most essential when predicting the future C cycle at high latitudes.

## Methods

### Site description of sampled subarctic lakes

The study was carried out in six subarctic lakes located in the Stordalen catchment, northern Sweden (68°N, 19°E), during 2010 (Fig. S1). The 15 km^2^ catchment includes alpine tundra terrain dominated by heaths and dwarf shrubs (*e.g. Empetrum hermaphroditum, Vaccinium sp. and Betula nana*) at high altitudes (770–600 m a.s.l.) and sub-alpine terrain at low altitudes (360–600 m a.s.l.) covered with mountain birch forest (*Betula pubescens* ssp. *Czerepanovii*) and peatlands (*Sphagnum* mosses or *Ericaceae* shrubs in the bog parts and *Eriophorium* in the fen parts) The catchment is located in the zone of discontinuous permafrost^32^ and the mires contain areas of palsa^33^. Mean annual air temperature for 2000–2009 was 0.6 ± 0.4 °C and the coldest and warmest months were February (−9.5 ± 3.1 °C) and July (12.5 ± 1.2 °C), respectively. The average annual total precipitation for the same period was 340 ± 56 mm. All climatological data was recorded at Abisko Scientific Station, Sweden. General information about the lakes are given in Table S1.

### GIS analyses

Areas of the lakes were obtained by digitalizing an orthophoto (1 m pixel resolution) using the software package Arc GIS 9.3.1 (ESRI, U.S.). Lake volumes were determined from interpolations of integrated GPS and echo sounding depth measurements (m52i , Lowrance, U.S.) and shoreline points randomly chosen from the orthophoto given a depth value of 0 m. Altogether were 2733 points used for the interpolations divided on 6 lakes (between 115 to 1370 points per lake depending on the lake size). Winter depth and volumes were determined by subtraction of the ice volumes covering the lakes. The interpolations were performed in the Arc GIS 9.3.1 geostatistical analysis package using the ordinary Kriging method. Maximum depths were confirmed by manual depths measurements.

### Water sampling and chemical analyses

The partial pressure of CO_2_ was measured hourly from a raft, 0.5 m sub-surface, at the deepest part of each lake, throughout the ice-free season in 2010. We used Vaisala CARBOCAP Carbon Dioxide Transmitters GMT 222 (Vaisala Oyj., Finland) infrared gas analyzers (IRGA) as described by Johnson *et al.*^34^. Measurements of CH_4_ fluxes were performed using floating chambers^35^. Two types of chambers were used in each lake, one type which collected the total flux (ebullition and diffusive fluxes) of CH_4_ and one with a underwater shield preventing the chambers from collecting CH_4_ bubbles. Measurements were carried out during two 48 h periods each month from June to August. The chambers (12 to 22 per lake depending on lake size) were arranged in transects in order to cover all different depth zones. Two chambers for measurements of diffusive fluxes were placed in each surveyed lake of which one at the deepest zone and the other at shallow waters. Temperature was measured in ten minutes intervals by HoBo temperature loggers (Onset Computer Cooperation, U.S.) in all lake outlets during the ice-free season. In addition, water grab samples (for CO_2_, CH_4,_ DIC and DOC analyses) were taken in each lake before and after the ice break-up in all lakes. When the lakes were ice-covered, samples were collected from three locations (deep, intermediate and shallow depth) and at each location we sampled at 0.5 m below the ice, at 0.5 m above the sediment surface (if total depth exceeded 3 m) and half way to the bottom (if the total depth exceeded 4.5 m) or else only sampled at two depths. For details see Karlsson *et al.*^36^.

All CH_4_ and DIC samples were analyzed in the headspace using a gas chromatograph (Clarus 500, Perkin Elmer Inc.) following Lundin *et al.*^37^. The grab samples for *p*CO_2_ of the water were measured with a headspace equilibrium technique^38^, using an infrared gas analyzer (EGM 4, PP-systems Inc., U.S.). DOC was analyzed after filtration (0.45 μm sterile filter, Filtropur S, Sarstedt AG & Co., Germany) and acidification (100 μL 20% HCl to 50 mL filtrate) by high temperature catalytic oxidation (HTCO) using a Shimadzu TOC-V CPH analyzer (Shimadzu Corporation, Japan).

### Post processing of data and gas flux calculations

The outputs of the Vaisala IRGAs were corrected for temperature and pressure following Johnson *et al.*^34^. Corrected values were then calibrated against standard gases measured for each individual set of IRGA and logger. Standard gas measurements were preformed both before and after the field season (R^2^ = 0.999). The water concentration of CO_2_ was calculated from partial pressures by using Henry’s law, knowing the temperature dependency of Henry’s constant (*K*_*h*_) ,the temperature of the solution and the volume relationship between liquid and gas phases^39^.

We estimated the momentum diffusive CO_2_ flux between water surface and atmosphere, using Fick’s law and wind dependent piston velocities given by Cole and Caraco^40^. Wind speed was measured (location showed in Fig. S1) with ultrasonic anemometer (Metek USA-1; METEK Gmbh., Germany), installed 7.5 m above the ground^41^.

Fluxes of CH_4_ into the floating chambers were calculated as done according to Bastviken *et al.*^35^. Sometimes, the calculated ebullition flux exceeded the diffusive flux, i.e. chamber concentrations were higher than the equilibrium concentration in water, which result in CH_4_ uptake. In those cases, fluxes were estimated by linear mass balance calculations.

Ice-free season CO_2_ emissions were calculated as the product of fluxes and lake area integrated over time. Emissions of CH_4_ were calculated product of spatial mean fluxes and lake areas integrated over time. Lake ice-free season was integrated from the day the first observed open water (May 21) to when all lakes were ice-covered (October 22), which gives an estimated ice-free season of 154 days. Emission of CO_2_ and CH_4_ at ice break-up in the six sampled lakes was determined as the difference in amount of CO_2_ and CH_4_ between the sampling occasion under spring ice and the first open water sampling after ice break-up^36^. Only lakes with a maximum depth above 1.5 m were assumed to accumulate gases during the winter season since shallower lakes freeze solid during winters.

### Sediment dating and C burial rates calculations

The sediment cores were collected in April 2011 (five lakes) and 2013 (one lake) from the lake ice using a HTH-Kajak (Pylonex Termokonsult, Sweden) gravity corer^42^. In total, we collected ten cores, divided between the six lakes depending on lake size and morphology. All sediment cores were sectioned directly in the field into 1 cm slices, transferred into polypropylene containers (4K 100, Nolato Cerbo AB, Sweden), then transported back to the laboratory within the same day and stored frozen at −20 ^o^C. All samples were freeze dried, ground by hand and homogenized prior to analysis.

Sediment C contents were measured using a Carlo Erba EA 1108 elemental analyzer (University of California Davis stable isotope facility, Davis, California) and for one batch of sediments by using an IL550 TOC analyzer (Hach-Lange, GmbH, Germany). We determined the content of inorganic C (carbonates) in the sediments as the mass loss after acid fumigation^43^.

Establishing the chronology and sedimentation rate of the sediments was based on ^210^Pb dating. This technique uses the vertical distribution of excess or unsupported ^210^Pb (T_1/2_ = 22.3 years) to establish accurate and precise chronologies of sedimentary deposits accumulated over the past 100–150 years^44^,^45^. ^210^Pb activities were determined by measuring the activities of its granddaughter ^210^Po, assumed to be in secular equilibrium with its parent nuclide in the sediment sample. ^210^Po analyses were performed according with the methodology described by Sanchez-Cabeza *et al.*^46^ that consists on the complete dissolution of the aliquot samples by microwave digestion and its deposition on silver discs. The isotope ^209^Po was used as a tracer for yield determination. Po sources were counted using Ortec (U.S.) ULTRA-AS Ion-Implanted-Silicon Charged-Particle Detectors (Model U-020-450-AS). Excess ^210^Pb was assessed by subtracting the ^210^Pb activities at depth from the total ^210^Pb.

The sediment burial rates were determined using the Constant Flux – Constant Sedimentation (CF:CS) model^47^, applied from the sediment depths where the ^210^Pb activity decreased monotonically with increased depth (≥2.5 cm depth). Notably, this selection generates estimates of sedimentation rates into older sediments and thus, provides rates representative for inputs to sediment layers where decomposition is progressing at a very slow rate^48^. Long-term C burial rates were calculated by multiplying the inferred sediment burial rates with the measured C concentration of each core. Only one core had estimated ^210^Pb inventories above 2.2 kBq m^−2^, an inventory observed for peat cores from the Stordalen mire^49^ indicating that; i) the effect of sediment focusing on the estimated burial rates was low; ii) correcting for sediment focusing would increase C burial rates for most of the cores. The C burial rates presented in the study, which were not corrected for sediment focusing, are thus conservative estimates for most of the cores.

### Meta analysis and statistics

We compiled listed and plotted data from published and peer reviewed journal papers and governmental reports. Plotted data was compiled by using the free software Plot Digitizer 2.6.4 (www.plotdigitizer.sourceforge.net). We compiled separate annual emission (Table S2) and sediment burial (Table S3) data but also paired data (Table S4). We defined the lakes boreal or subarctic – arctic based on the definition by Callaghan *et al.*^50^. Lakes located at the borderline between temperate and boreal environments or located within areas heavily influenced by agricultural activities were not considered. Therefore, we did not select data from lakes located in northern USA (except for Alaska) or southern Scandinavia. Further, since significant sediment respiration occurs within the first top cm of sediments, sediment C burial estimates not corrected for that might be largely overestimated^51^. Thus, we did not include C burial estimates based on the first top cm only. If multiple estimates from the same lake existed and the quality of work was considered equal, the newest estimate was used.

The differences between subarctic – arctic and boreal C emission and burial data from the listed literature (Table S2 and S3) were tested by one-way ANOVAs. Differences between subarctic – arctic and boreal paired emission:burial ratios (new data and previous publications, altogether 89 boreal and 10 subarctic – arctic lakes lakes, see Table S4) and its dependency of lake sizes or DOC concentrations was tested by ANCOVA. We tested the regression slopes (Fig. 1) by ANCOVA. We preformed logarithmic transformations when needed to achieve normal distributions. All statistical variance tests were carried out in the open source software RStudio 0.97.316 (RStudio Inc, U.S.). We estimated the present C fluxes in lakes at 63°–90° N by multiplying the total lakes areas^52^ with average emission values for subarctic – arctic lakes (Table S2). We express all our result as arithmetic means ± standard deviations, if not otherwise stated.

## Additional Information

**How to cite this article**: Lundin, E. J. *et al.* Large difference in carbon emission-burial balances between boreal and arctic lakes. *Sci. Rep.* **5**, 14248; doi: 10.1038/srep14248 (2015).

## Supplementary Material

Supplementary Information
