# Supplementary material for: Large difference in carbon emission – burial balances between boreal and arctic lakes
Source: Sci Rep. 2015 Sep 15;5:14248. doi: 10.1038/srep14248 (PMC4642577; doi:10.1038/srep14248)
Supplement: Supplementary Information [file srep14248-s1.doc]

**Large difference in carbon emission - burial balances between boreal and arctic lakes**

**Supporting Information**

E. J. Lundin^1,6*^, J. Klaminder^2^, D. Bastviken^3^, C. Olid^2^, S. V. Hansson^4,5^, J. Karlsson^6^

^1^ Department of Environmental Science and Analytical Chemistry (ACES), Stockholm University, SE-106 91 Stockholm, Sweden

^2^ Department of Ecology and Environmental Science, Umeå University, SE-90187 Umeå, Sweden

^3^ Department of Thematic Studies*–*Environmental Change, Linköping University, SE-58183 Linköping, Sweden

^4^ Université de Toulouse; INP, UPS; EcoLab; ENSAT, Avenue de l’Agrobiopole,

31326 Castanet Tolosan, France

^5^ CNRS; EcoLab; 31326 Castanet Tolosan, France

^6^ Climate Impacts Research Centre (CIRC), Department of Ecology and Environmental Science,Umeå University, SE-981 07 Abisko, Sweden

^*^Corresponding author: [erik.lundin@aces.su.se](mailto:erik.lundin@aces.su.se)

**Figure S1**

The location of the subarctic study lakes in the Stordalen catchment in northern Sweden. The theoretical catchment pour point is through the outlet of lake L1. The green star shows the location of the wind measurements. Coordinates are given in decimal degrees (WGS 84). The figure was generated using the software package Arc GIS 9.3.1 (ESRI, U.S.).

**Figure S2**

Fluxes of CO_2_ and CH_4_ from the six subarctic lakes in northern Sweden during the ice-free season 2010 (ice thaw fluxes not shown). Positive fluxes means emission of C, while negative means uptake. Fluxes of CH_4_ are expressed as spatial averages. The whiskers indicate the first standard deviation of averages.

**Figure S1**


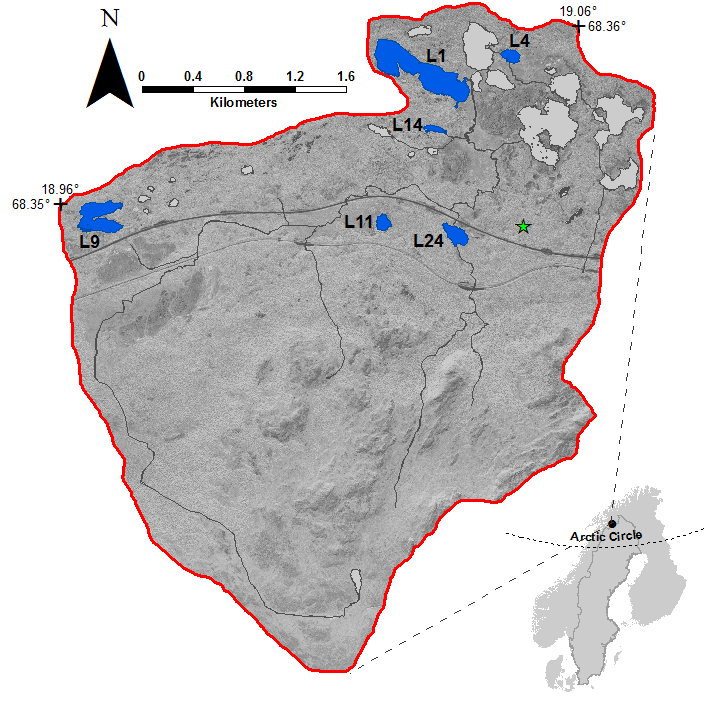


**Figure S2**

**
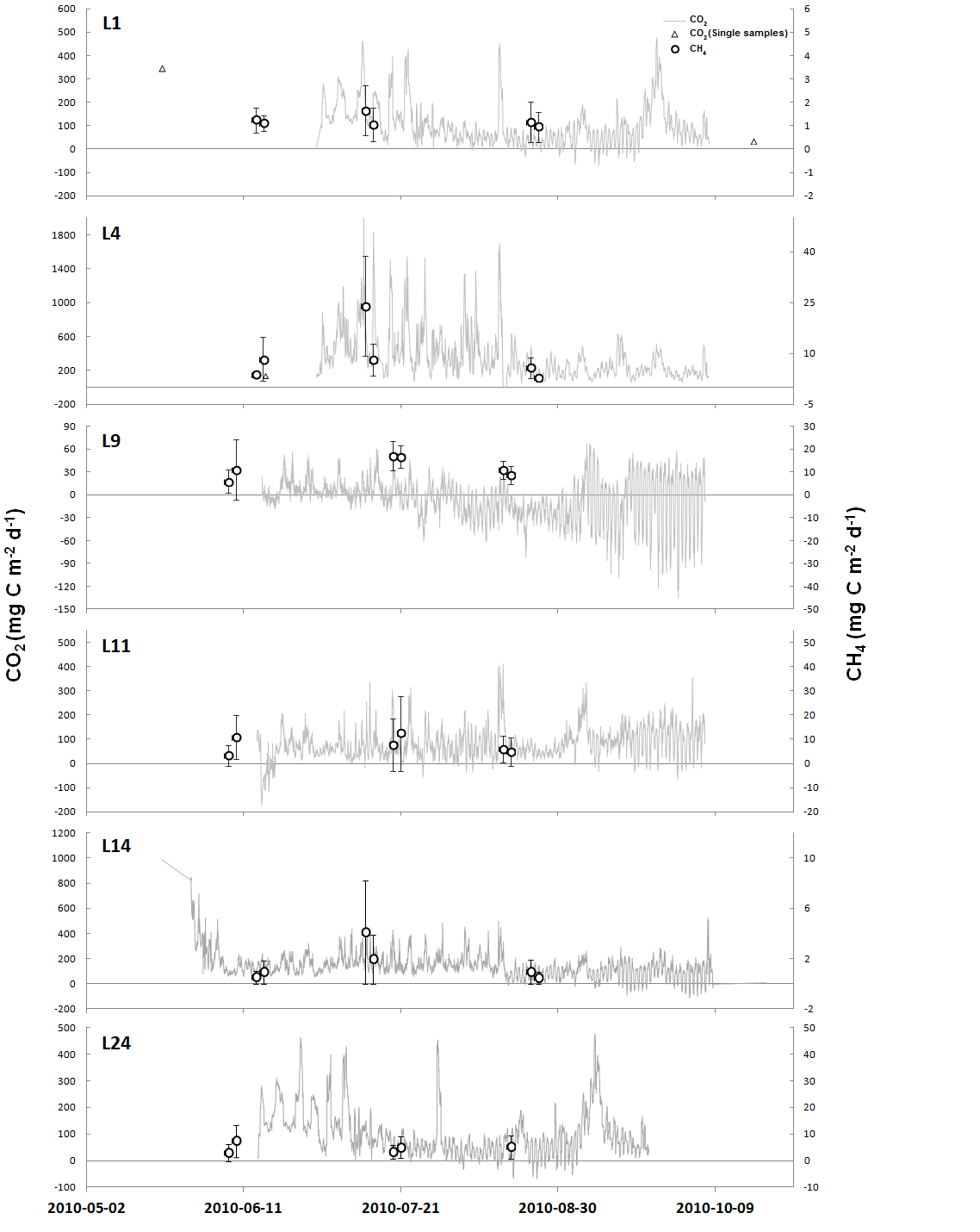
**

**Table S1.** Lake characteristics and annual fluxes of C in the subarctic lakes in northern Sweden during ice-free season 2010. The C burial was predominately consisting of organic C since no inorganic C was detected in the sediments. Concentrations are expressed as summer season averages ± one standard deviation.

| **Lake** | **Area** | **Volume** | **Mean depth** | **Max depth** | **DIC concentration** | **CH_4_** **concentration** | **DOC concentration** | **Inorganic C burial** | **Organic C burial** | **C emission** |
| --- | --- | --- | --- | --- | --- | --- | --- | --- | --- | --- |
|  | **(m^2^)** | **(m^3^)** | **(m)** | **(m)** | **(mg C L^-1^)** | **(mg C L^-1^)** | **(mg C L^-1^)** | **(g C m^-2^ yr^-1^)** | **(g C m^-2^ yr^-1^)** | **(g C m^-2^ yr^-1^)** |
| **L1** | 138500 | 320700 | 2.3 | 8.5 | 3.8 ± 1.4 | 0.03 ± 0.02 | 8.5 ± 1.5 | - | 12 | 30 |
| **L4** | 11100 | 12500 | 1.1 | 3.5 | 6.3 ± 1.5 | 0.05 ± 0.04 | 9.4 ± 1.8 | - | 25 | 54 |
| **L9** | 57300 | 105000 | 1.9 | 4.5 | 4.3 ± 0.6 | 0.05 ± 0.06 | 6.7 ± 0.6 | - | 11 | 5 |
| **L11** | 10400 | 4500 | 0.4 | 1 | 3.1 ± 0.5 | 0.03 ± 0.01 | 9.7 ± 1.1 | - | 18 | 14 |
| **L14** | 6100 | 8100 | 1.3 | 2.5 | 4.5 ± 0.6 | 0.02 ± 0.01 | 7.6 ± 0.4 | - | 18 | 28 |
| **L24** | 20100 | 27000 | 1.3 | 2.5 | 5.2 ± 1.4 | 0.03 ± 0.01 | 5.4 ± 1.7 | - | 5 | 12 |

**Table S2.** C emission data from earlier publications and this study.

| **Publication** | **Location** | **Number of lakes** | **C emission** | **Class** |
| --- | --- | --- | --- | --- |
|  |  |  | **(g C m^-2^** **yr^-1^)** |  |
| Brothers et al. (2012) | Quebec, Canada | 13 | 8-125 | Boreal |
| Einola et al. (2011) | Finland | 4 | 25-68 | Boreal |
| Einola et al. (2011) | Finland | 1 | 77 | Boreal |
| Åberg et al. (2004) | Sweden | 2 | 21-24 | Boreal |
| Kortelainen et al. (2013) | Finland | 71 | 2-230 | Boreal |
| Karlsson et al. (2010) | Sweden | 1 | 32 | Subarctic |
| Eugster et al. (2003) | Alaska, U.S. | 1 | 11 | Arctic |
| Jansson et al. (2008) | Sweden | 12 | 1-23 | Subarctic |
| Juutinen et al. (2013) | Finland | 1 | 15* | Subarctic |
| Karlsson unpublished | Sweden | 2 | 13-14 | Subarctic |
| Kling et al. (1992) | Alaska, U.S. | 6 | 9-72* | Arctic |
| Lundin et al. (2013) | Sweden | 21 | -3-150* | Subarctic |
| Repo et al. (2007) | Siberia, Russia | 3 | 24-66* | Subarctic |
| Rocher et al. in preparation | Sweden | 1 | 6* | Subarctic |
| This study | Sweden | 6 | 5-54* | Subarctic |

*** = Studies including both CO_2_ and CH_4_ in the C emission estimate**

**Table S3.** Sediment C burial data from earlier publications and this study.

| **Publication** | **Location** | **Number of lakes** | **C burial** | **Class** |
| --- | --- | --- | --- | --- |
|  |  |  | **(g C m^-2^ yr^-1^)** |  |
| Dillon & Molot (1997) | Ontario, Canada | 7 | 2.2-10.1 | Boreal |
| Einola et al. (2011) | Finland | 4 | 1.0-6.0 | Boreal |
| Einola et al. (2011), Pajunen (2000) | Finland | 1 | 2.8 | Boreal |
| Ferland et al. (2012) | Quebec, Canada | 13 | 1.2-8.0 | Boreal |
| Flower et al. (1995) | Siberia, Russia | 1 | 2.6 | Boreal |
| Pajunen (2000) | Finland | 31 | 1.1-13.0 | Boreal |
| Squires et al. (2006) | Alberta, Canada | 4 | 40-90 | Boreal |
| Whalen & Cornwell (1985) | Alaska, U.S. | 1 | 2.6 | Arctic |
| Anderson et al. (2009) | Greenland | 11 | 1.4-11.6 | Arctic |
| Willemse & Törnquist (1999) | Greenland | 2 | 4.9-7.2 | Arctic |
| Juutinen et al. (2013) | Finland | 1 | 7.7 | Subarctic |
| Klaminder et al. (2010) | Sweden | 1 | 0.6 | Subarctic |
| Kokfelt et al. (2010) | Sweden | 1 | 15.3 | Subarctic |
| This study | Sweden | 6 | 5.3-24.6 | Subarctic |

**Table S4.** Lakes from where both C emission and C burial have been estimated.

| **Publication** | **Area** | **Number of lakes** | **C emission** | **C burial** | **Class** | **Size** |
| --- | --- | --- | --- | --- | --- | --- |
|  |  |  | **(g C m^-2^ yr^-1^)** | **(g C m^-2^ yr^-1^)** |  | **(km^2^)** |
| Einola et al (2011), Pajunen (2000) | Finland | 1 | 77 | 3 | Boreal | 0.04 |
| Einola et al. (2011) | Finland | 4 | 25-68 | 0.3-6 | Boreal | 0.5-44 |
| Ferland et al. (2012), Brothers et al. (2012) | Quebec, Canada | 13 | 8-183 | 1-8 | Boreal | 0.1-25 |
| Kortelainen et al. 2013 | Finland | 71 | 2-230 | 0.2-9 | Boreal | - |
| Eugester et al. 2003, Whalen & Cornwell (1985) | Alaska, U.S. | 1 | 11 | 3 | Arctic | 1.5 |
| Jansson et al. (2008), Klaminder et al. (2010) | Sweden | 1 | 4 | 1 | Subarctic | 0.01 |
| Juutinen et al. (2013) | Finland | 1 | 15 ^*^ | 8 | Subarctic | 1.6 |
| Kokfelt et al. (2010), Karlsson et al. (2010) | Sweden | 1 | 32 | 15 | Subarctic | 0.02 |
| This study | Sweden | 6 | 5-54 ^*^ | 5-25 | Subarctic | 0.01-0.1 |

*** = Studies including both CO_2_ and CH_4_ in the C emission estimate. All other studies only include CO_2_ emission.**

**Supporting Information References**

Anderson Nj, D'andrea W, Fritz Sc (2009) Holocene carbon burial by lakes in SW Greenland. *Global Change Biology,* **15**, 2590-2598.

Brothers Sm, Prairie Yt, Del Giorgio Pa (2012) Benthic and pelagic sources of carbon dioxide in boreal lakes and a young reservoir (Eastmain-1) in eastern Canada. *Global Biogeochemical Cycles,* **26**, 10.

Dillon Pj, Molot La (1997) Dissolved organic and inorganic carbon mass balances in central Ontario lakes. *Biogeochemistry,* **36**, 29-42.

Einola E, Rantakari M, Kankaala P *et al.* (2011) Carbon pools and fluxes in a chain of five boreal lakes: A dry and wet year comparison. *Journal of Geophysical Research-Biogeosciences,* **116**, 13.

Eugster W, Kling G, Jonas T, Mcfadden Jp, Wuest A, Macintyre S, Chapin Fs (2003) CO2 exchange between air and water in an Arctic Alaskan and midlatitude Swiss lake: Importance of convective mixing. *Journal of Geophysical Research-Atmospheres,* **108**, 20.

Ferland Me, Del Giorgio Pa, Teodoru Cr, Prairie Yt (2012) Long-term C accumulation and total C stocks in boreal lakes in northern Quebec. *Global Biogeochemical Cycles,* **26**, 10.

Flower Rj, Mackay Aw, Rose Nl *et al.* (1995) Sedimentary records of recent environmental-change in lake baikal, Siberia. *Holocene,* **5**, 323-327.

Jansson M, Hickler T, Jonsson A, Karlsson J (2008) Links between terrestrial primary production and lake mineralization and CO_2_ emission in a climate gradient in subarctic Sweden. *Ecosystems*, DOI: 10.1007/s10021-10008-19127-10022.

Juutinen S, Väliranta M, Kuutti V *et al.* (2013) Short-term and long-term cabon dynamics in a northern peatland- stream-lake continuum - a catchment approach. *Journal of Geophysical Research-Biogeosciences,* **118**, 171-183.

Karlsson J, Christensen Tr, Crill P *et al.* (2010) Quantifying the relative importance of lake emissions in the carbon budget of a subarctic catchment. *Journal of Geophysical Research-Biogeosciences,* **115**, 6.

Klaminder J, Hammarlund D, Kokfelt U, Vonk Je, Bigler C (2010) Lead Contamination of Subarctic Lakes and Its Response to Reduced Atmospheric Fallout: Can the Recovery Process Be Counteracted by the Ongoing Climate Change? *Environmental Science & Technology,* **44**, 2335-2340.

Kling Gw, Kipphut Gw, Miller Mc (1992) The flux of CO_2_ and CH_4_ from lakes and rivers in Arctic Alaska. *Hydrobiologia,* **240**, 23-36.

Kokfelt U, Reuss N, Struyf E *et al.* (2010) Wetland development, permafrost history and nutrient cycling inferred from late Holocene peat and lake sediment records in subarctic Sweden. *Journal of Paleolimnology,* **44**, 327-342.

Kortelainen P, Rantakari M, Pajunen H *et al.* (2013) Carbon evasion/accumulation ratio in boreal lakes is linked to nitrogen. *Global Biogeochemical Cycles,* **27**, 363-374.

Lundin E, J., Giesler R, Persson A, Thompson M, S., Karlsson J (2013) Integrating carbon emissions from lakes and streams in a subarctic catchment. *Journal of Geophysical Research-Biogeosciences,* **118**, 1-8.

Pajunen H (2000) Lake sediments: their carbon store and related accumulation rates. In: *Carbon in Finnish Lake Sediments, Geological Survey of Finland, Special Paper 29.* (ed Pajunen H) pp Page. Kuopio, Finland.

Repo Me, Huttunen Jt, Naumov Av, Chichulin Av, Lapshina Ed, Bleuten W, Martikainen Pj (2007) Release of CO2 and CH4 from small wetland lakes in western Siberia. *Tellus Series B-Chemical and Physical Meteorology,* **59**, 788-796.

Squires Mm, Mazzucchi D, Devito Kj (2006) Carbon burial and infill rates in small Western Boreal lakes: physical factors affecting carbon storage. *Canadian Journal of Fisheries and Aquatic Sciences,* **63**, 711-720.

Whalen Sc, Cornwell Jc (1985) Nitrogen, phosphorus, and organic-carbon cycling in an arctic lake *Canadian Journal of Fisheries and Aquatic Sciences,* **42**, 797-808.

Willemse Nw, Tornqvist Te (1999) Holocene century-scale temperature variability from West Greenland lake records. *Geology,* **27**, 580-584.

Åberg J, Bergström Ak, Algesten G, Söderback K, Jansson M (2004) A comparison of the carbon balances of a natural lake (L. Örtrasket) and a hydroelectric reservoir (L. Skinnmuddselet) in northern Sweden. *Water Research,* **38**, 531-538.
